# How far would you go to sleep better at high altitude?

**DOI:** 10.1093/sleep/zsac277

**Published:** 2022-11-22

**Authors:** Esteban A Moya, Omar A Mesarwi

**Affiliations:** Section of Physiology, Division of Pulmonary, Critical Care, and Sleep Medicine, Department of Medicine, University of California San Diego, La Jolla, CA, USA; Section of Physiology, Division of Pulmonary, Critical Care, and Sleep Medicine, Department of Medicine, University of California San Diego, La Jolla, CA, USA

Patients with obesity may show disturbances in ventilation during wakefulness and sleep, and may develop sleep-disordered breathing (e.g. obesity hypoventilation syndrome and/or obstructive sleep apnea) [1–3], potentially resulting in changes in both daytime and nighttime partial pressure of CO_2_ and O_2_. These changes in arterial CO_2_ and O_2_ cause the activation of ventilatory chemoreflexes aiming to restore physiologic values. Thus, disruption of the ventilatory chemoreflexes, or their interaction, may contribute to the progression of breathing-related diseases, highlighting the importance of understanding the mechanisms of control of breathing in respiratory diseases. Animal models are useful to study these mechanisms, since they mimic features observed in such conditions. For instance, a study performed in a model of obese mice showed that these rodents had lower pH and higher arterial partial pressure of CO_2_, as well as decreased oxyhemoglobin saturations in awake and sleep stages, when compared to lean mice [4], nicely recapitulating findings in humans.

In a recent publication in *Sleep*, Kim et al. [5] studied diet-induced obese mice to test the hypothesis that acute, severe hypoxia would destabilize breathing during sleep, but that concurrent exposure to a small but constant amount of CO_2_ (isocapnia) would improve sleep-disordered breathing, presumably by stimulating ventilation. The authors performed polysomnograms in these mice, measuring ventilatory parameters in non-rapid eye movement (NREM) sleep during the administration of 10% oxygen alone (poikilocapnic hypoxia), and separately, 10% oxygen with 3% CO_2_ (isocapnic hypoxia). The authors found that obese mice exposed to isocapnic hypoxia increase minute ventilation, driven both by increases in respiratory rate and tidal volume, when compared to mice exposed to normoxia or poikilocapnic hypoxia. Moreover, they applied Poincaré analysis to study the short- and long-term respiratory variability in each condition (with short-term variability represented as distance from the line of unity on the Poincaré plot, and long-term variability represented as the degree of scatter on the line of unity). The authors found that, compared to normoxia, isocapnic hypoxia resulted in more long-term respiratory variability, whereas poikilocapnic hypoxia did not differ from normoxia. On the other hand, poikilocapnic hypoxia increased short-term respiratory variability compared to normoxia, but no effect of isocapnic hypoxia was observed. Thus, in obese mice, poikilocapnic hypoxia destabilizes breathing on a breath-to-breath basis, an effect abolished by the addition of CO_2_. Short-term respiratory variability is a critical concept in considering implications of high-altitude hypoxia, since hypoxia-induced changes in ventilation may acutely cause periodic breathing and Cheyne–Stokes breathing patterns.

The authors additionally examined effects of these conditions on sleep apnea severity and sleep architecture. They found that obese mice exposed to poikilocapnic hypoxia had a significant increase in the apnea index when compared to normoxic mice, with all such apneas being central. Given the finding that poikilocapnic hypoxia destabilizes breathing, this is perhaps unsurprising. However, this effect was abrogated by the addition of CO_2_ in isocapnic hypoxia; in these mice, the apnea index was not different from mice in normoxia. Accordingly, both hypoxic conditions were associated with reductions in mean oxyhemoglobin saturation, but in isocapnic hypoxia, saturations were higher overall than in poikilocapnic hypoxia, likely due to fewer respiratory events. Finally, NREM sleep time was lower in isocapnic hypoxia, and REM sleep time was lower in both hypoxic conditions (and in fact, poikilocapnic hypoxia completely eliminated REM sleep). The implication of these findings is that isocapnic hypoxic may cause mice to spend more time awake, though data exploring this effect in more detail (i.e. hypnographic data) are not available.

Exposure to acute hypoxia triggers the hypoxic ventilatory response (HVR), initiated by activation of O_2_-sensitive peripheral chemoreceptors located in the carotid body. In poikilocapnic conditions, hypoxia-induced hyperventilation results in a reduction in arterial partial pressure of CO_2_, thereby causing a deactivation of the CO_2_-sensitive central chemoreceptors, and a blunted HVR response. On the other hand, the intentional addition of CO_2_ to inspired hypoxic gas, in order to maintain isocapnia, will produce a higher increase in ventilation driven only by the O_2_ chemoreceptors, without the inhibitory effect of low CO_2_ levels. Thus, the increased ventilation and higher O_2_ saturations observed in obese mice during isocapnic conditions (relative to poikilocapnic) as demonstrated by Kim et al. [5] is consistent with our general understanding of the mechanisms of control of breathing.

These observations, combined with the increase in central respiratory events in poikilocapnic hypoxia, nicely mirror human data from those residing at high altitude [6–8]. Highlanders tend to have more severe sleep-disordered breathing than lowlanders, driven by more severe central sleep apnea [9]. The increased ventilation in obese mice induced by isocapnic hypoxia (and not inhibited by low arterial CO_2_ levels, as it is putatively in poikilocapnic hypoxia) may maintain the mice in a state of “activated breathing” avoiding the occurrence of central apnea events by avoiding proximity to the apneic CO_2_ threshold. The obvious questions from this line of research are: What specific role does obesity play in this paradigm? Are there potential therapeutic applications of this research? Might one expect acute and/or chronic alterations in ventilation and sleep apnea severity by, for instance, occasional rebreathing techniques, which tend to increase arterial partial pressure of CO_2_? How could this be applied practically, and how might this compare to the administration of a medication which might serve to stimulate ventilation, such as acetazolamide?

The work by Kim et al. encourages us to propose new, exciting questions in the field, but also demonstrates that nothing in life is free: Although ventilatory stability, oxygenation, and sleep apnea severity all improved in isocapnic hypoxia relative to poikilocapnic hypoxia, total sleep time appeared substantially reduced, and this merits further investigation. What does sleep look like in these mice, and what comparisons can be made to humans at altitude? Studies performed in Andeans show that a blunted HVR response is associated with lower daytime oxygen saturation. And that a decreased ventilatory response to high levels of CO_2_ (hypercapnic ventilatory response) observed in Andeans is associated with more severe nocturnal hypoxemia [10]. Moreover, hemodilution in Andeans to improve excessive erythrocytosis results in surprising effects on sleep apnea and hypoxemia severity at high altitude [11]. The work of Kim et al. [5] highlights the need for fuller exploration of possible off-target and unintended effects. Hypoxia at high altitude modulates several important sleep quality parameters, including total sleep time, sleep efficiency, and number of central and obstructive apnea events [12]. And ventilatory acclimatization to hypoxia, after days of exposure, improves sleep quality [13, 14]. The contributions of the control of breathing on sleep quality and breathing stability are not well understood, and merit further consideration.

Kim et al. [5] is highly commendable work, which provides useful data about the effects of poikilocapnic and isocapnic hypoxia on ventilation and sleep, and for the first time applies these concepts in an obese mouse model. These data will provide a useful basis to study further the associations between O_2_ and CO_2_ ventilatory responses and their interactions with sleep parameters, and eventually to elucidate the mechanisms underlying these interactions.
